# Machine-Learning Prediction of Health-Related Quality of Life Among Community-Dwelling Middle-Aged and Older Adults Living Alone: A Secondary Analysis of the 2022 Korea Health Panel

**DOI:** 10.3390/healthcare14121669

**Published:** 2026-06-11

**Authors:** Sunkyung Cha, Miran Jung, Geun Myun Kim, Seong Kwang Kim

**Affiliations:** 1 Department of Nursing Science, Sun Moon University, Asan 31460, Republic of Korea; skc0701@hanmail.net; 2 Department of Nursing, Baekseok University, Cheonan 31065, Republic of Korea; 3 Department of Nursing, Kangwon National University, Wonju 26403, Republic of Korea; gmkim@kangwon.ac.kr (G.M.K.); tjdrhkd141@kangwon.ac.kr (S.K.K.)

**Keywords:** quality of life, health status, machine learning

## Abstract

**Highlights:**

**What are the main findings?**

- A nationally representative dataset study was used to develop and benchmark an explainable machine-learning (ML) model of health-related quality of life (HRQoL) for community-dwelling Korean adults aged ≥40 years living alone.
- The model outperformed the mean baseline, and SHapley Additive exPlanations (SHAP) analysis ranked subjective health, age, and walking time as leading predictors for this specific population.

**What are the implications of the main findings?**

- The top predictors of physical activity such as walking time and regular exercise and unmet medical needs, are modifiable, making them concrete candidate targets for future intervention research in adults living alone.

**Abstract:**

**Background/Objectives**: Because the numbers of middle-aged and older adults living alone in Korea have substantially increased, which warrants greater attention to their health-related quality of life. Therefore, we aimed to develop a predictive model for the health-related quality of life among community-dwelling middle-aged and older adults living alone. **Methods**: Using 2022 Korea Health Panel Survey data, 1313 participants with complete EQ-5D component data were analyzed. All candidate predictors were entered into benchmarked models without pre-model feature selection. Preprocessing and 5-fold cross-validated hyperparameter tuning were conducted within the training data. Final performance was evaluated on a held-out test set, and the selected model was interpreted using SHAP. **Results**: XGBoost had the lowest training cross-validated RMSE and was selected as the final explainable model. On the test set, it showed moderate performance (R^2^ = 0.373, MAE = 0.070, RMSE = 0.096), outperforming the mean baseline model (RMSE = 0.121) but remaining comparable with other top-performing models. Predictions were within absolute errors of 0.05 and 0.10 for 45.6% and 76.4% of participants, respectively. SHAP ranked subjective health, age, walking time, need for care, and monthly household income as the five highest-ranked predictors. Other highly ranked predictors included unmet medical needs, total annual out-of-pocket expenditure, disability, anxiety, and regular exercise. **Conclusions**: These findings may inform targeted interventions and support strategies, although external validation and longitudinal studies are needed to confirm generalizability and causal relationships.

## 1. Introduction

One-person households are becoming increasingly prevalent as the Korean population ages. Their numbers increased from 4.17 million in 2010 to 7.34 million in 2023—about a 1.7-fold increase—representing 33.6% of all households [1]. The proportion of inhabitants aged ≥80 years is particularly high, indicating that living alone has become a mainstream household profile in Korean society [1]. This trend is projected to continue until 2040, with steady growth, particularly among middle-aged single-person households (aged 40–60 years). Although the definition of middle age varies, it generally refers to individuals aged 40 to 65 years [2].

In South Korea, national youth policies and municipal ordinances, including those of Seoul and Gyeonggi Province, define youth as individuals aged ≤39 years and middle-aged and older aged adults are defined as being aged ≥40 years in Korean demographic and public health studies [3]. We adopted these age ranges. Studying adults aged ≥40 years enables the identification of key predictors that will contribute to the health-related quality of life (HRQoL) before people reach an advanced age, and potential heterogeneity across this broad age range is addressed through age-subgroup sensitivity analyses comparing participants aged 40–64 years with those aged ≥65 years.

Consequently, community-dwelling middle-aged and older adults living alone (aged ≥40 years) have emerged as key populations in community care planning and public health, and improving their HRQoL has become an increasingly important priority.

Korea has traditionally relied on family-based caregiving. Therefore, older adults living alone might be vulnerable to deficits in emotional support and daily care, which can adversely affect their HRQoL. Previous studies have reported that social isolation among individuals living alone is associated with depression, reduced quality of life, and increased mortality risk. When chronic illness or care needs arise, the responsibility for accessing long-term care often shifts toward both individuals and society [4,5]. Therefore, identifying determinants of HRQoL and developing targeted interventions are essential for effective public health policy implementation.

Most studies have focused on adults aged ≥65 years, and have compared them to individuals living in multi-person households. These studies have consistently found a tendency towards a lower HRQoL among single-, than multi-person households that declines over time [5]. Psychological and socioeconomic factors, such as income, self-esteem, and depressive symptoms are associated with life satisfaction and the HRQoL [6]. However, households comprising a single inhabitant aged 40 years have not been analyzed in detail as distinct population groups. Understanding the HRQoL determinants at this transitional stage is important because early health vulnerabilities and socioeconomic disadvantages might accumulate and influence health trajectories later in life.

However, many of these studies have relied primarily on traditional regression-based approaches that assume linear relationships between variables. In complex public health contexts, factors influencing HRQoL—such as socioeconomic status, health behaviors, mental health, and healthcare utilization—often interact in nonlinear and multidimensional ways that conventional statistical models might not be able to fully capture.

A recent decision-tree study of 2019 Korea Health Panel Survey (KHPS) data identified subjective health perception, chronic disease status, income, and age as key determinants of quality of life among single-person households with older adult [7]. However, a single-algorithm approach was applied in that study without multi-model benchmarking, and the contributions of the individual predictors were not determined. Multi-algorithm benchmarking using explainable artificial intelligence techniques such as SHapley Additive exPlanations (SHAP)-based predictor interpretation in this population has never been applied as far as we can ascertain. Hence, we aimed to address this knowledge gap.

Recent advances in machine learning (ML) have provided alternative analytical approaches that address some limitations of traditional statistical models. ML algorithms are can model nonlinear relationships and complex interactions among many predictors without requiring strict assumptions about data distribution.

Accordingly, this study applies a ML approach to complement traditional regression methods by modeling nonlinear associations among demographic, socioeconomic, and health-related variables and identifying priority factors associated with HRQoL. Specifically, several ML algorithms were benchmarked, then the model with the best performance was selected based on cross-validated Root Mean Square Error (RMSE) for a final interpretation using SHAP.

SHAP can improve the interpretability of ML models by quantifying the contribution of individual predictors to model outcomes. The utility of SHAP across diverse healthcare contexts includes pressure injury prediction in intensive care units [8], opioid overdose death risk stratification [9], and QoL prediction in arthritis [10]. This approach enables transparent identification of key determinants of HRQoL that are policy-relevant.

Therefore, we aimed to develop and benchmark ML prediction models of HRQoL among community-dwelling, middle-aged, and older adults living alone and to interpret the best-performing model using SHAP. Specifically, we aimed to identify and prioritize key predictors that influence the HRQoL by interpreting SHAP-based models to provide evidence to inform targeted public health strategies and screening tools to improve HRQoL in this rapidly growing population.

## 2. Materials and Methods

### 2.1. Study Design

This study aimed to develop, benchmark, and internally validate ML prediction models for the EQ-5D index of community-dwelling adults aged ≥40 years living alone. The secondary objective was to identify factors that contribute to model predictions. Our analyses focused on sample-based prediction and predictor prioritization.

### 2.2. Data Collection

This study utilized data from the 2022 wave of the KHPS Annual Data, Version 2.3, 2019–2022 [11]. The KHPS is a nationally representative survey jointly conducted by the Korea Institute for Health and Social Affairs and the National Health Insurance Service. This government-approved statistical survey (Approval No. 920012) was reviewed and approved by the Institutional Review Board of Korea Institute for Health and Social Affairs. All data were rendered innominate according to the Personal Information Protection Act. The KHPS systematically collects comprehensive information on disease status, healthcare utilization, medical expenditures, and health-related behaviors.

The survey was a two-stage, stratified cluster sampling design based on the 2016 Population and Housing Census. We stratified 17 metropolitan cities and provinces during the first stage, followed by the administrative units of Dong, Eup, and Myeon during the second stage. Enumeration districts were then selected using probability proportional to size sampling. Data were collected via in-person computer-assisted personal interviews (CAPI) conducted by trained interviewers. Data reliability was enhanced by encouraging respondents to verify their information using National Health Insurance Service claims data, household account books, and medical receipts.

Study variables were categorized into the following domains: (1) sociodemographic characteristics, including sex, age, marital status, education level, graduation status, region, housing type, and type of health security; (2) economic characteristics, including employment status, monthly household income, and public pension recipients; (3) health status and behavior including disability, chronic disease, need for care, regular exercise, walking time (min/day), lifetime smoking history, average alcohol consumption per drinking occasion, perceived stress, subjective health, depressive symptoms, anxiety, and suicidal ideation; and (4) healthcare access and use, expenditure, and insurance variables, including medical aid benefits, usual source of care, unmet medical and dental care, annual out-of-pocket expenditures, perceived medical expense burden, private health insurance enrollment, and private health insurance policy counts.

The KHPS provides cross-sectional and longitudinal survey weights for design-based population inference. However, we did not incorporate survey weights because the primary aim was to develop and compare predictive models to identify sample-based predictor contributions rather than to estimate population parameters. Therefore, all descriptive statistics, model performance estimates, and SHAP-based predictor rankings should be interpreted as unweighted sample-based results.

### 2.3. Study Participants

We analyzed samples derived from a raw dataset by sequentially applying the following inclusion criteria. Data acquired from inhabitants aged ≥40 years at the time of analysis were defined as middle-aged and older adults and were included in the target population. This cut-off reflected the age ranges applied in Korean demographic and public health research [2], in which middle age is typically defined as ages between 40–64 years. This has been applied to adults living alone when examining HRQoL. Only single-person households were included in the final analysis to enable in-depth assessments of changes in household structures and related health issues, only single-person households (households consisting of one member) were included in the final analysis. After applying the inclusion criteria and excluding data without EQ-5D component items, we final analyzed data from 1313 samples. Because the study included a broad age range spanning middle-aged and older adults, age was retained as a continuous predictor. We then compared age-subgroup sensitivity between groups aged 40–64 and ≥65 years.

### 2.4. Data Preprocessing

Data were preprocessed and analyzed using the pandas, Scikit-learn, XGBoost, and SHAP packages in Python 3.10. Three KHPS SAS files, namely household data (d_hh), member-change data (d_id), and individual data (d_ind) were imported and merged using HHID and PIDWON. Single-person households were identified using FAM_N = 1.

The EQ-5D index was computed from five EQ-5D dimensions using the Korean EQ-5D valuation algorithm [12]. Subjective health is presented descriptively using the original survey coding, where 1 = very good and 5 = very poor. Subjective health was reverse-coded such that higher values indicated better subjective health to develop models. Walking time (min/day) was derived from the KHPS walking-time variable, which records the average number of minutes walked per day; participants reporting no walking were assigned 0 min according to the survey logic. Average alcohol consumption per drinking occasion was coded as 0 for participants who reported lifetime abstinence or no alcohol consumption during the past year.

Special missing code −9, indicating “do not know/no response,” was treated as missing. For annual out-of-pocket expenditure variables, missing values reflecting no corresponding healthcare utilization were treated as structural zeros in the primary analysis. These variables included total annual out-of-pocket expenditure and annual emergency, inpatient, outpatient, and prescription out-of-pocket expenditures. However, special missing codes indicating “do not know/no response” were not treated as structural zero values. To assess whether the structural-zero decision influenced model performance, we conducted a sensitivity analysis in which missing cost-variable values were handled using median imputation instead of structural-zero coding.

Missing values in continuous predictors were imputed using the median within the model-preprocessing pipeline. Missing indicators were added to continuous variables when applicable. Categorical predictors were not entered as original survey-coded numeric values. Instead, categorical variables were explicitly treated as categorical predictors and one-hot encoded. Missing categorical values were retained as separate missing categories where applicable. For Ridge regression and Support Vector Regression, continuous variables were additionally standardized within the training-data preprocessing pipeline.

### 2.5. Training/Test Data Split and Preprocessing

The analytical dataset was split into training and held-out test sets in an 80:20 ratio, with a fixed random state of 42. As the outcome was continuous, the EQ-5D index was divided into quantile-based bins, and stratified splitting preserved the outcome distribution across the training and test sets. The final split was 1050 and 263 in the training and held-out test sets, respectively before imputation, one-hot encoding, standardization, hyperparameter tuning, and model fitting. All preprocessing steps included only the training data and the held-out test set was applied only to the final performance evaluation.

### 2.6. Model Development and Benchmarking

Pre-model features were not selected. All candidate predictors were retained and entered into the benchmark prediction models. This approach avoided excluding predictors that might have weak linear associations with the EQ-5D index but meaningful nonlinear or interaction-based contributions to ML models.

The following were benchmarked: the mean baseline dummy model, ridge regression, Decision Tree, Random Forest, Gradient Boosting, Histogram-based Gradient Boosting, Support Vector Regression, and XGBoost. These models were selected to represent the major algorithmic families commonly used in regression analyses, including linear models, single decision trees, tree-based ensembles based on bagging and boosting, and kernel-based regressions. The mean baseline dummy model was included as a lower-bound reference to confirm whether the prediction models performed significantly better than the sample mean. Ridge regression was included as a linear baseline model that was robust to multicollinearity through regularization, allowing direct comparisons with nonlinear models. The Decision Tree was included as a benchmark for single-tree performance. Random Forest represented bagging-based tree ensembles, whereas Gradient and Histogram-based Gradient Boosting represented sequential boosting-based tree ensembles. Kernel-based nonlinear regression approaches were compared using Support Vector Regression. XGBoost was included as a scalable gradient-boosted tree algorithm with built-in regularization and missing-value handling that has consistently delivered strong predictive performance on tabular data [13]. Given the relatively modest sample size (*n* = 1313) and the structured, tabular nature of the predictors, deep neural network-based models, which typically benefit from large-scale data, were not included in the benchmark comparison.

Hyperparameter tuning within the training set was achieved using 5-fold cross-validation. The optimization criterion was the cross-validated RMSE. Supplementary Table S1 shows the hyperparameter grids and selected hyperparameters. The models were ranked according to the cross-validated RMSE of the training set. XGBoost achieved the lowest training cross-validated RMSE and was therefore selected as the final explainable ML model. The held-out test set was used only for final evaluation of the tuned models.

### 2.7. Model Evaluation

Model performance was evaluated using mean absolute error (MAE), mean squared error (MSE), root mean squared error (RMSE), median absolute error (MedAE), and the coefficient of determination (R^2^). The proportions of predictions within absolute error thresholds of 0.05 and 0.10 in the original EQ-5D scale were calculated to provide interpretable error-threshold metrics.

The cross-validated performance of benchmarked models was summarized as means ± standard deviation (SD) among five training folds. The performance of the final model was evaluated using the held-out test set. For the final XGBoost model, 95% confidence intervals (CIs) were estimated using 1000 bootstrap resamples of the held-out test set.

### 2.8. Model Interpretation

We interpreted the final XGBoost model using SHAP analysis [14], which provides additive feature attributes for model predictions and is frequently used to explain complex ML models. TreeExplainer is designed for tree-based models and provides efficient SHAP value estimations for tree ensembles [14].

Because the categorical variables were one-hot encoded, the SHAP values were first calculated at the encoded feature level. For presentation at the original predictor level, the SHAP values for the one-hot encoded features belonging to the same original predictor were aggregated. Predictors were ranked according to mean absolute SHAP values. The top 20 predictors are displayed in the main SHAP bar plot and in a SHAP summary plot for readability, and the full SHAP ranking is retained for supplementary reporting. We generated SHAP dependence plots for the five highest-ranked predictors in the main text, and additional dependence plots for predictors ranked 6–10 that were generated for supplementary reporting.

The SHAP dependence plots were interpreted as exploratory prediction profiles and were not included as formal tests of nonlinear threshold effects. No causal interpretation was made from SHAP values.

### 2.9. Sensitivity Analysis

We determined the robustness of structural-zero coding for cost variables by comparing primary and sensitivity analyses in which missing cost-variable values were handled using median imputation within the model preprocessing pipeline. We also addressed potential heterogeneity across a broad age range, using an age-subgroup sensitivity analysis. The final XGBoost model trained on the full training set was applied separately to participants aged 40–64 years and those aged ≥65 years in the held-out test set. The model performance and SHAP-ranked predictors were also compared between the two age subgroups.

## 3. Results

### 3.1. Descriptive Statistics of Key Variables

The final analytical sample of data was derived from 1313 community-dwelling adults aged 40 years who lived alone. Table 1 shows selected characteristics of the analytical sample, and Supplementary Table S2 provides full descriptive statistics for all candidate predictors.

The mean age was 73.02 ± 11.52 (median 75.00; range, 40.00–97.00) years. The sample comprised 356 (27.1%) men and 957 (72.9%) women. The mean EQ-5D index was 0.89 ± 0.12 (median, 0.91; range −0.06–1.00). Monthly household incomes averaged 164.94 ± 134.16 (median, 119.38) per 10,000 KRW. The mean walking time was 50.96 ± 45.91 min/day, with a median of 40.00 min/day.

Subjective health was assessed using the original survey coding for descriptive statistics. The most frequent category was fair subjective health (*n* = 526; 40.1%), followed by poor (*n* = 388; 29.6%) and good health (*n* = 333; 25.4%). Elementary school was the most prevalent education level (*n* = 454, 34.6%), followed by high school (*n* = 303, 23.1%) and middle school (*n* = 240, 18.3%). Overall, 625 participants (47.6%) were economically inactive. Chronic disease was reported by 1109 participants (84.5%), and 102 participants (7.8%) reported a need for care. Unmet medical needs were reported by 215 participants (16.4%), and 766 participants (58.3%) had private health insurance.

Supplementary Figure S1 shows histograms and boxplots of key continuous variables, including age, monthly household income, EQ-5D index, and walking time (min/day).

### 3.2. Model Benchmark Performance

Table 2 shows the benchmark performance of the regression and ML models, ordered by training cross-validated RMSE. XGBoost achieved the lowest training cross-validated RMSE (0.087 ± 0.009) and the highest training cross-validated R^2^ (0.439 ± 0.038); therefore, it was selected as the final explainable ML model. On the held-out test set, XGBoost achieved MAE = 0.070, RMSE = 0.096, and R^2^ = 0.373, and its performance was broadly comparable to that of other top-performing models. The mean baseline dummy model performed substantially worse than Ridge regression, Histogram-based Gradient Boosting, Random Forest, and XGBoost. These benchmark results indicate that the final XGBoost model had moderate predictive capacity and performed substantially better than the mean baseline model, while its held-out test performance was similar to that of other top-performing regression and ML models.

### 3.3. Final XGBoost Model Performance

Table 3 shows the final held-out test performance of the XGBoost model, with bootstrap 95% CIs. The model achieved MAE = 0.070 (95% CI: 0.063–0.079), MSE = 0.009 (95% CI: 0.007–0.012), RMSE = 0.096 (95% CI: 0.082–0.112), MedAE = 0.056 (95% CI: 0.048–0.062), and R^2^ = 0.373 (95% CI: 0.284–0.457). The proportion of predictions within an absolute error of 0.05 was 0.456 (95% CI: 0.399–0.517), and the proportion within an absolute error of 0.10 was 0.764 (95% CI: 0.715–0.810).

### 3.4. Interpretation of Results Using SHAP

**Figure 1:**
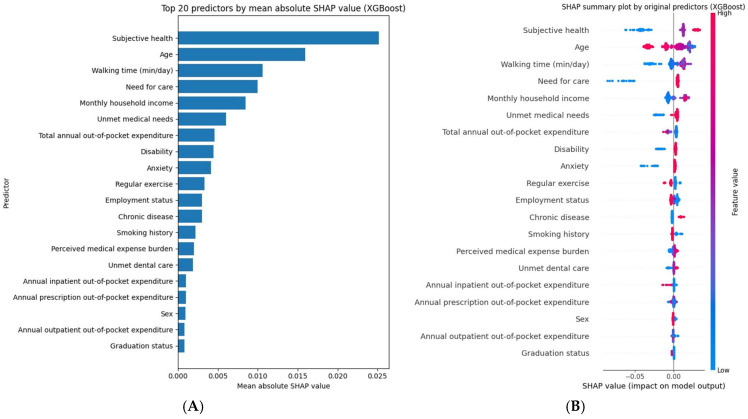
SHAP-based interpretation of the final XGBoost model. (**A**) Bar plot showing the top 20 predictors ranked by mean absolute SHAP value. Larger values indicate greater average contribution to model predictions. (**B**) SHAP summary plot showing the direction and magnitude of each predictor’s contribution to the predicted EQ-5D index in the held-out test set. The main figure displays the top 20 predictors for readability; the full SHAP ranking of all candidate predictors is provided in Supplementary Table S3. Each point represents one participant. For categorical predictors, colors represent encoded category values and should not be interpreted as continuous gradients unless the original variable was ordinal.

Figure 1 shows a SHAP-based interpretation of the final XGBoost model. The bar plot ranks the predictors according to mean absolute SHAP values, and the summary plot shows the direction and magnitude of the predictor contributions to the predicted EQ-5D index in the held-out test set. Supplementary Table S3 shows the SHAP rankings of all candidate predictors.

The five highest-ranked predictors were subjective health, age, walking time (min/day), need for care, and monthly household income. The next highest ranked predictors were unmet medical needs, total annual out-of-pocket expenditure, disability, anxiety, and regular exercise. Thus, subjective health had the largest average contribution to model predictions, followed by age and walking time (min/day). Anxiety was among the top 10 predictors, whereas depressive symptoms did not appear among the top 20 predictors in the final XGBoost model. Supplementary Figure S2 presents an encoded-feature SHAP summary plot.

**Figure 2:**
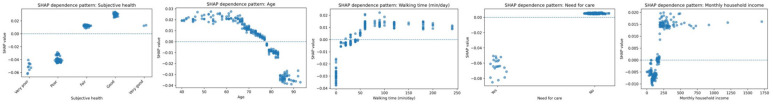
SHAP dependence patterns for the five highest-ranked predictors in the final XGBoost model. The plots are shown for the five predictors with the largest mean absolute SHAP values: subjective health, age, walking time (min/day), need for care, and monthly household income. Each point represents one participant in the held-out test set. The x-axis shows the observed predictor value, and the y-axis shows the SHAP value, indicating the predictor’s contribution to the model output. These plots are presented to describe exploratory prediction patterns and should not be interpreted as statistically confirmed threshold effects. Additional SHAP dependence plots for other top-ranked predictors are provided in Supplementary Figure S3.

Figure 2 shows SHAP dependence plots for the five highest-ranked predictors and describes exploratory prediction patterns in the held-out test set. Higher subjective health scores contributed positively to predicted EQ-5D index values, whereas lower subjective health scores contributed negatively. Older age generally contributed negatively. Longer walking time and higher monthly household income tended to contribute positively to model predictions across parts of their observed ranges. Need for care showed a category-specific pattern, with participants reporting a need for care generally contributing lower predicted EQ-5D index values. These dependence profiles should be interpreted as exploratory model-based patterns rather than statistically confirmed threshold effects. Supplementary Figure S3 shows SHAP dependence plots for predictors ranked 6th to 10th.

### 3.5. Sensitivity Analysis Results

Supplementary Table S4 shows the sensitivity analysis of structural-zero coding for cost variables. The primary analysis using structural-zero coding yielded MAE = 0.070, RMSE = 0.096, and R^2^ = 0.373. The sensitivity analysis using median imputation for cost-variable missing values yielded MAE = 0.070, RMSE = 0.096, and R^2^ = 0.377. These results suggest that model performance was robust to the alternative handling of cost-variable missingness.

Supplementary Table S5 shows the results of the age-subgroup sensitivity analysis. Among 53 participants aged 40–64 years in the held-out test set, the final XGBoost model achieved MAE = 0.038, RMSE = 0.050, and R^2^ = 0.665. Among participants aged ≥65 years (*n* = 210), the model achieved MAE = 0.078, RMSE = 0.104, and R^2^ = 0.296. Predictive performance was higher in the subgroup aged 40–64 years than in those aged ≥65 years, although the test-set sample of the younger subgroup was smaller.

Supplementary Table S6 presents the top ten SHAP-ranked predictors according to age subgroup. Subjective health, age, walking time (min/day), need for care, monthly household income, and unmet medical needs were important factors in both age groups. Chronic disease and employment status were relatively more prominent among the subgroup aged 40–64 years, whereas disability and regular exercise were relatively more prominent among participants aged ≥65 years.

## 4. Discussion

This study focused on key factors influencing HRQoL among middle-aged and older adults living alone in Korea based on data from the 2022 KHPS. We explored the impact of these variables on the HRQoL using the final XGBoost model. Our findings provide valuable evidence for future strategies and support policies for middle-aged and older single-person households because the KHPS is a nationally representative public dataset.

The SHAP analysis of the XGBoost model provided detailed insights into the magnitude and direction of the effects of the most influential features. The five most important variables affecting the HRQoL were subjective health status, age, walking daily, need for care, and monthly household income. Among the 20 most contributory variables identified in Figure 1, most were categorized under health status and health behaviors as well as healthcare access, use, and expenditure. This likely reflects the distinctive characteristics of the KHPS dataset, which systematically includes comprehensive information on disease status, healthcare use, medical expenditure, and health-related behaviors.

Our findings reinforce previous results, highlighting the importance of subjective health status as a comprehensive indicator reflecting physical, psychological, and social dimensions of health [2,7,15,16,17]. Consistent with earlier findings, unmet medical needs and economic activity were associated with the HRQoL [15]. A lower HRQoL is associated with being female [15] and economically inactive [18,19]. Employment status might be related to better access to socioeconomic resources and well-being [20,21], whereas incomplete schooling might reflect a socioeconomic disadvantage. Mental health factors such as anxiety, depressive symptoms, and suicidal ideation have been identified as important correlates [2,15,18,22]. However, the present study associated anxiety only with the HRQoL. Moreover, the association between socioeconomic status, such as income level, and HRQoL has been consistently reported by others [21,23].

Individual feature values in the SHAP summary plot are represented in red and blue according to their magnitude and meaning. Features positioned further to the right and left respectively indicate increased and decreased model output. Clear visual color separation was observed for subjective health status, need for care, disability status, anxiety, presence of chronic diseases, unmet medical needs, perceived medical cost burden, regular exercise, smoking history, sex, and educational attainment. These results are consistent with previous findings indicating that higher HRQoL scores are associated with better subjective health, absence of chronic disease, and higher income levels [7,23,24,25].

A notable contribution of this study is the identification of nonlinear profiles using SHAP dependence plots that might not be fully captured by traditional regression models. The positive association between age and the HRQoL was relatively stable up to about the age of 65 years, followed by a progressively negative association with progressive aging. A lower monthly income was similarly associated with a lower HRQoL, whereas income above a certain threshold was associated with a higher HRQoL. Time spent walking every day that exceeded ~ 50 min was positively associated with HRQoL [25]. However, this study was exploratory in nature and requires prospective validation.

The SHAP analysis highlighted potentially modifiable factors that might serve as targets for intervention. Variables such as time spent walking, anxiety, and regular exercise can be leveraged in targeted interventions to improve physical and mental aspects of HRQoL. Examples include community-based walking programs tailored to middle-aged and older adults living alone and screening-based interventions using anxiety assessment tools. Variables related to healthcare access and financial burden, including unmet medical needs, unmet dental care, annual out-of-pocket expenditures, by inpatient, and outpatients, total out-of-pocket expenditures, and perceived medical expense burden represent modifiable domains. Policy-level strategies could include reducing unmet medical and dental care needs, establishing administrative support systems tailored to single-person households, and providing financial assistance or counseling for healthcare-related expenditures, including inpatient and outpatient cost-sharing.

However, we acknowledge that this study has limitations. Our model predicts HRQoL based on associated factors, and causal relationships cannot be inferred due to its cross-sectional design. Therefore, the associations determined herein should be interpreted as correlational, rather than causal. Our model performed moderately in terms of internal validation. However, we did not externally validate our model, which might limit generalizability to other populations or settings. External validation is essential for broader implementation or policy applications. The relatively modest sample and large number of categorical variables introduced the possibility of overfitting, which is a statistical limitation. Future research should address these limitations by clarifying the causal mechanisms and strengthening the evidence base by integrating objective biomarkers such as electronic medical records (EMRs) or wearable device data, and by validating the model among diverse populations.

## 5. Conclusions

The rapid increase in the number of middle-aged and older adults living alone underscores a growing need to address their HRQoL. This study identified an ML-based predictive model for estimating the EQ-5D index among Korean adults aged ≥40 years living alone in a community and internally validated. The predictive performance of the final XGBoost model was moderate. The SHAP analysis indicated that subjective health status, age, amount of time spent walking per day, need for care, monthly household income, and unmet medical needs were among the most important predictors. The key predictive variables fell largely within the domains of health status and behaviors, as well as healthcare access, use, and expenditure. These findings suggest potential policy and intervention strategies, including the development of regular exercise programs, screening-based anxiety management interventions, and the establishment of administrative and financial support systems to reduce healthcare-related expenditures.

However, the cross-sectional design precludes causal inference, and the relatively small sample combined with several categorical variables present statistical limitations. The findings are specific to middle-aged and older Korean adults aged ≥40 years living alone, and external validation is required before generalizing to other populations or settings. Future studies should use longitudinal data to investigate causal relationships and validate this model among diverse populations and settings.

## Figures and Tables

**Table 1:** Selected characteristics of the analytic sample (N = 1313).

Characteristics	Category	Mean ± SD	Median [IQR]	Range	*n* (%)
Age (years)		73.02 ± 11.52	75.00 [67.00–82.00]	40.00–97.00	—
Monthly household income (10,000 KRW)		164.94 ± 134.16	119.38 [84.67–200.42]	0.00–1708.33	—
Walking time (min/day)		50.96 ± 45.91	40.00 [20.00–60.00]	0.00–300.00	—
EQ-5D index		0.89 ± 0.12	0.91 [0.82–1.00]	−0.06–1.00	—
Sex	Male	—	—	—	356 (27.1)
	Female	—	—	—	957 (72.9)
Subjective health	Very good	—	—	—	28 (2.1)
	Good	—	—	—	333 (25.4)
	Fair	—	—	—	526 (40.1)
	Poor	—	—	—	388 (29.6)
	Very poor	—	—	—	38 (2.9)
Education level	No formal education	—	—	—	155 (11.8)
	Elementary school	—	—	—	454 (34.6)
	Middle school	—	—	—	240 (18.3)
	High school	—	—	—	303 (23.1)
	University/college	—	—	—	138 (10.5)
	Graduate school	—	—	—	23 (1.8)
Employment status	Wage worker	—	—	—	326 (24.8)
	Public/support work program participant	—	—	—	186 (14.2)
	Employer	—	—	—	10 (0.8)
	Self-employed	—	—	—	166 (12.6)
	Not economically active	—	—	—	625 (47.6)
Need for care	Yes	—	—	—	102 (7.8)
	No	—	—	—	1211 (92.2)
Chronic disease	Yes	—	—	—	1109 (84.5)
	No	—	—	—	204 (15.5)
Disability	Yes	—	—	—	171 (13.0)
	No	—	—	—	1142 (87.0)
Unmet medical needs	Yes	—	—	—	215 (16.4)
	No	—	—	—	1086 (82.7)
	No need for medical care	—	—	—	12 (0.9)
Private health insurance enrollment	Yes	—	—	—	766 (58.3)
	No	—	—	—	541 (41.2)
	Do not know	—	—	—	6 (0.5)

Values are presented as mean ± SD, median [IQR], range, or *n* (%). Table 1 presents selected demographic, socioeconomic, health-related, and model-relevant characteristics of the analytic sample; full descriptive statistics for all candidate predictors are provided in Supplementary Table S2. All descriptive statistics are unweighted. Subjective health is presented using the original survey coding in this table; for model development, subjective health was reverse-coded so that higher values indicated better subjective health.

**Table 2:** Benchmark performance of machine-learning and regression models.

Model	Training CV MAE (Mean ± SD)	Training CV RMSE (Mean ± SD)	Training CV R^2^ (Mean ± SD)	Test MAE	Test RMSE	Test R^2^	Test Proportion with |Error| ≤ 0.05	Test Proportion with |Error| ≤ 0.10
XGBoost	0.067 ± 0.004	0.087 ± 0.009	0.439 ± 0.038	0.070	0.096	0.373	0.456	0.764
Ridge regression	0.068 ± 0.004	0.088 ± 0.008	0.427 ± 0.023	0.070	0.096	0.375	0.449	0.757
Histogram-based Gradient Boosting	0.067 ± 0.004	0.088 ± 0.008	0.424 ± 0.032	0.071	0.096	0.380	0.464	0.772
Support Vector Regression	0.069 ± 0.004	0.089 ± 0.009	0.417 ± 0.032	0.071	0.096	0.369	0.464	0.753
Gradient Boosting	0.068 ± 0.004	0.089 ± 0.007	0.415 ± 0.025	0.072	0.096	0.372	0.445	0.768
Random Forest	0.067 ± 0.003	0.089 ± 0.008	0.412 ± 0.033	0.071	0.096	0.373	0.479	0.772
Decision Tree	0.075 ± 0.004	0.098 ± 0.009	0.281 ± 0.057	0.083	0.109	0.199	0.414	0.719
Mean baseline (dummy model)	0.097 ± 0.003	0.117 ± 0.010	−0.007 ± 0.009	0.097	0.121	−0.002	0.285	0.388

Models are sorted by cross-validated RMSE in the training set. Hyperparameter tuning was performed within the training set using 5-fold cross-validation. XGBoost achieved the lowest training cross-validated RMSE and was therefore selected as the final explainable machine-learning model. The held-out test set was used only for final performance evaluation.

**Table 3:** Test-set performance of the final XGBoost model with bootstrap 95% confidence intervals.

Metric	Point Estimate	95% Bootstrap CI
MAE	0.070	0.063–0.079
MSE	0.009	0.007–0.012
RMSE	0.096	0.082–0.112
MedAE	0.056	0.048–0.062
R^2^	0.373	0.284–0.457
Proportion with |error| ≤ 0.05	0.456	0.399–0.517
Proportion with |error| ≤ 0.10	0.764	0.715–0.810

CI, confidence interval; MAE, mean absolute error; MedAE, median absolute error; RMSE, root mean squared error. Confidence intervals were estimated using 1000 bootstrap resamples of the held-out test set. MAPE was not reported because the EQ-5D index is a bounded outcome and may include values close to zero.

## Data Availability

All data for this study are publicly available from the 2022 Korea Health Panel Survey (KHPS) at https://www.khp.re.kr:444/, accessed on 12 June 2025.

## Supplementary Materials

The following supporting information can be downloaded at: https://www.mdpi.com/article/10.3390/healthcare14121669/s1, Figure S1: Histograms and boxplots of key continuous variables; Figure S2: Encoded-feature SHAP summary plot for the final XGBoost model; Figure S3: Additional SHAP dependence plots for top-ranked predictors; Table S1: Hyperparameter grids and selected hyperparameters for benchmark models; Table S2: Full descriptive statistics of candidate predictors; Table S3: Full SHAP ranking of all candidate predictors in the final XGBoost model; Table S4: Sensitivity analysis for structural-zero treatment of cost variables; Table S5: Age-subgroup sensitivity analysis of final model performance; Table S6: Top 10 SHAP-ranked predictors by age subgroup.

## Abbreviations

The following abbreviations are used in this manuscript:

HRQoL	Health-related quality of life
KHPS	Korea health panel survey
RF	Random forest
SHAP	Shapley additive explanations
CAPI	Computer-assisted personal interviews
MAE	Mean absolute error
RMSE	Root mean squared error
MAPE	Mean absolute percentage error
