# Supplementary material for: Machine-Learning Prediction of Health-Related Quality of Life Among Community-Dwelling Middle-Aged and Older Adults Living Alone: A Secondary Analysis of the 2022 Korea Health Panel
Source: Healthcare (Basel). 2026 Jun 11;14(12):1669. doi: 10.3390/healthcare14121669 (PMC13299840; doi:10.3390/healthcare14121669)
Supplement: Supplementary file 1 [file healthcare-14-01669-s001.zip › healthcare-4300060-supplementary.pdf]

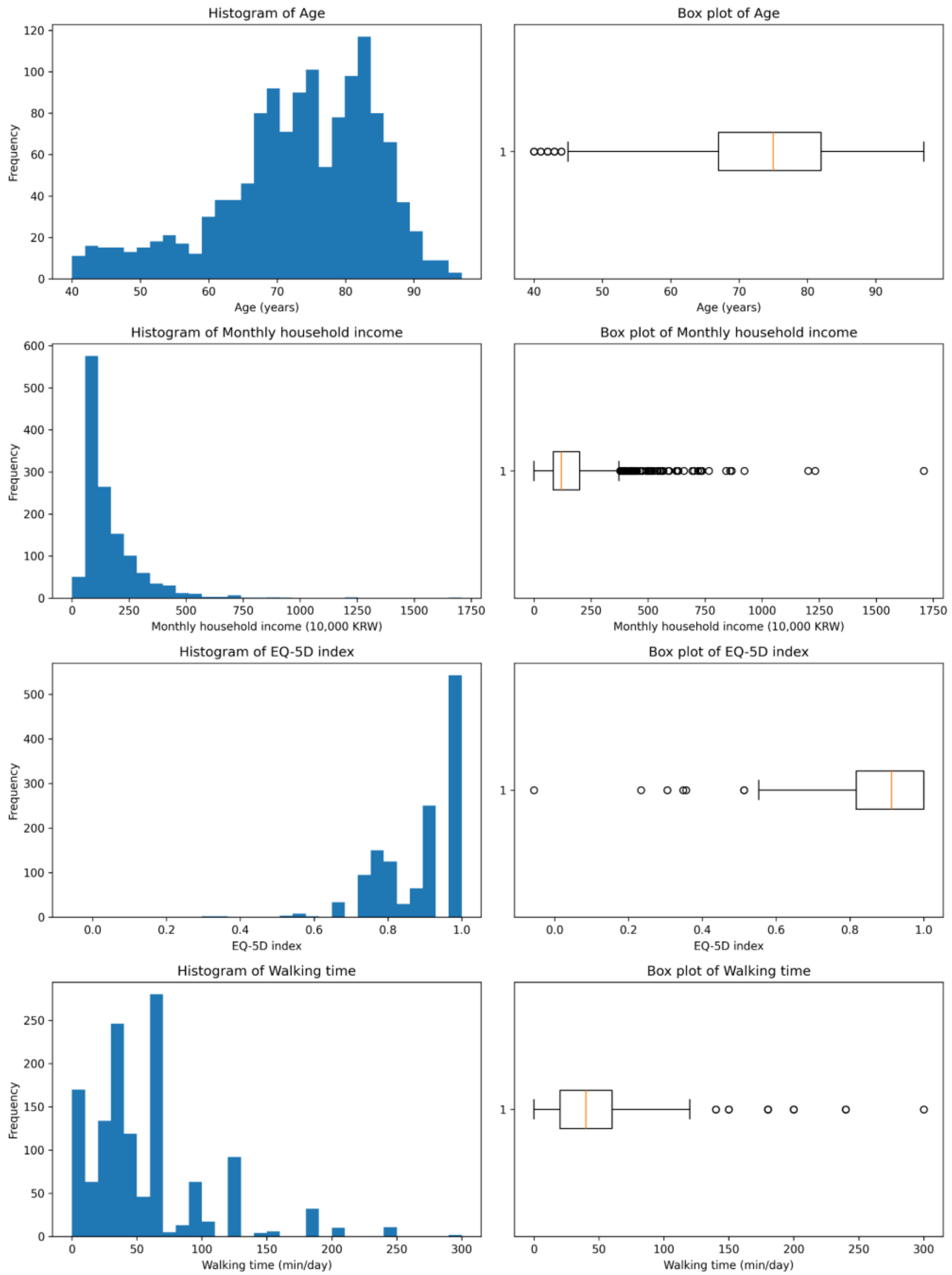

Supplementary Figure S1. Histograms and boxplots of key continuous variables

Caption: The figure shows the distributions of age, monthly household income, EQ-5D index, and walking time in the analytic sample.

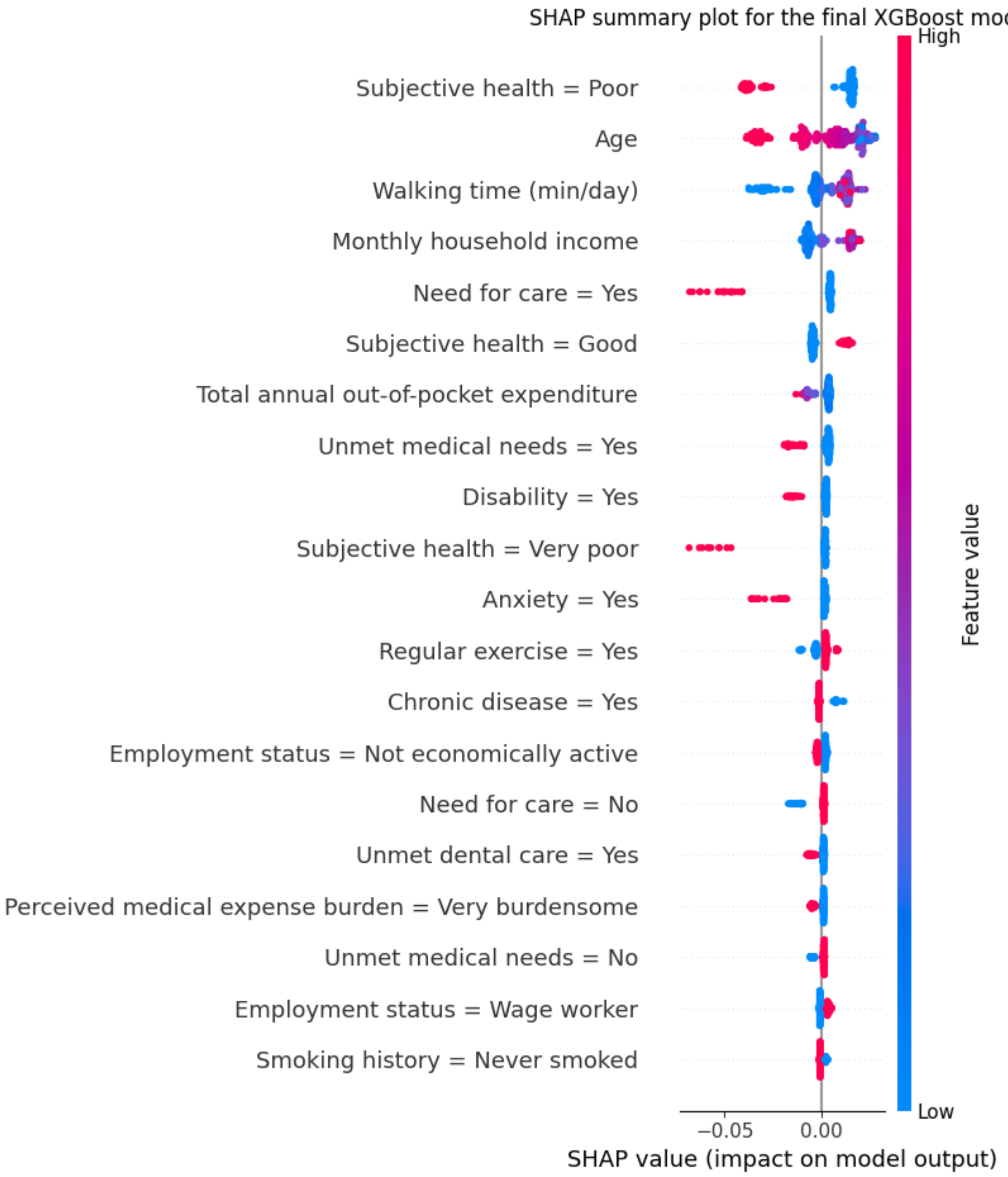

Supplementary Figure S2. Encoded-feature SHAP summary plot for the final XGBoost model.

Caption: This plot displays SHAP values at the encoded-feature level after one-hot encoding of categorical predictors. Each point represents one participant in the held-out test set. The x-axis shows the SHAP value, indicating the direction and magnitude of each encoded feature's contribution to the model output.

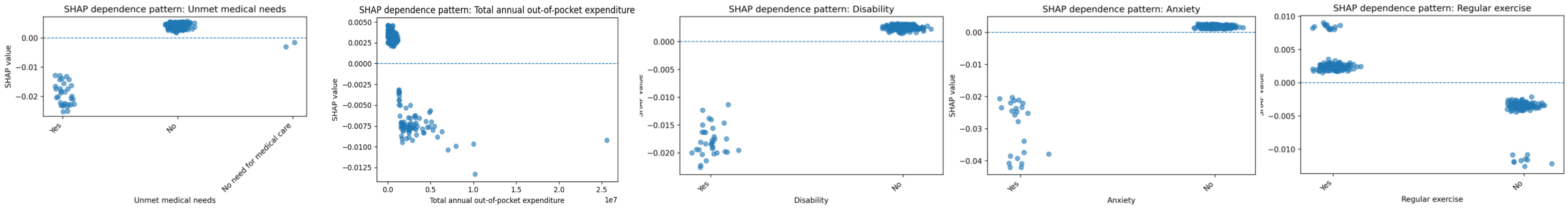

Supplementary Figure S3. Additional SHAP dependence plots for top-ranked predictors.

Caption: These plots show exploratory SHAP dependence patterns for predictors ranked 6th to 10th by mean absolute SHAP value in the final XGBoost model. The plots are provided to supplement the main-text dependence plots and should not be interpreted as statistically confirmed threshold effects.

Supplementary tables

Supplementary Table S1. Hyperparameter grids and selected hyperparameters for benchmark models

Model	Hyperparameter grid	Selected hyperparameters
Mean baseline (dummy model)	No tuning; strategy = mean	Mean prediction
Ridge regression	alpha = 0.1, 1.0, 10.0, 100.0	alpha = 100.0
Decision Tree	max_depth = 3, 5, 10, None; min_samples_leaf = 1, 2, 5, 10	max_depth = 5; min_samples_leaf = 10
Random Forest	n_estimators = 100, 200; max_depth = 10, 20, None; min_samples_split = 2, 5; min_samples_leaf = 1, 2, 5	n_estimators = 200; max_depth = 10; min_samples_split = 2; min_samples_leaf = 2
Gradient Boosting	n_estimators = 100, 200; learning_rate = 0.03, 0.05, 0.10; max_depth = 2, 3, 5	n_estimators = 100; learning_rate = 0.05; max_depth = 2
Histogram-based Gradient Boosting	max_iter = 100, 200; learning_rate = 0.03, 0.05, 0.10; max_leaf_nodes = 15, 31	max_iter = 100; learning_rate = 0.03; max_leaf_nodes = 15
Support Vector Regression	C = 0.1, 1, 10; epsilon = 0.01, 0.05, 0.10; kernel = RBF	C = 0.1; epsilon = 0.05; kernel = RBF
XGBoost	n_estimators = 100, 200; learning_rate = 0.03, 0.05, 0.10; max_depth = 2, 3, 5; subsample = 0.8, 1.0; colsample_bytree = 0.8, 1.0; reg_lambda = 1.0, 5.0	n_estimators = 200; learning_rate = 0.03; max_depth = 2; subsample = 0.8; colsample_bytree = 0.8; reg_lambda = 5.0

Supplementary Table S2. Full descriptive statistics of candidate predictors

Supplementary Table S2A. Continuous variables

Variable	N	Mean ± SD	Median [IQR]	Range
Age, years	1313	73.02 ± 11.52	75.00 [67.00–82.00]	40.00–97.00
Monthly household income, 10,000 KRW	1312	164.94 ± 134.16	119.38 [84.67–200.42]	0.00–1708.33
Walking time, min/day	1313	50.96 ± 45.91	40.00 [20.00–60.00]	0.00–300.00
Total annual out-of-pocket expenditure, KRW	1313	1,319,144.08 ± 1,949,505.18	654,180.00 [275,720.00–1,551,340.00]	0.00–25,590,663.00
Annual emergency out-of-pocket expenditure, KRW	1313	13,626.39 ± 71,231.15	0.00 [0.00–0.00]	0.00–1,298,990.00
Annual inpatient out-of-pocket expenditure, KRW	1313	360,582.06 ± 1,363,177.59	0.00 [0.00–0.00]	0.00–24,165,590.00
Annual outpatient out-of-pocket expenditure, KRW	1313	688,847.69 ± 993,078.41	319,900.00 [96,700.00–860,100.00]	0.00–9,250,600.00
Annual prescription out-of-pocket expenditure, KRW	1313	256,087.94 ± 286,174.81	186,500.00 [34,000.00–379,300.00]	0.00–3,509,200.00
Number of individual private health insurance policies	1313	1.45 ± 1.93	1.00 [0.00–2.00]	0.00–25.00
Number of household private health insurance policies	1313	1.45 ± 1.93	1.00 [0.00–2.00]	0.00–25.00
EQ-5D index	1313	0.89 ± 0.12	0.91 [0.82–1.00]	−0.06–1.00

Supplementary Table S2A note. Continuous variables are summarized using observed values before model-pipeline imputation. Out-of-pocket expenditure variables represent annual expenditures in Korean won.

Supplementary Table S2B. Categorical variables

Characteristics	Category	n (%)
Sex	Female	957 (72.9)
	Male	356 (27.1)
Marital status	Widowed	816 (62.1)
	Divorced	320 (24.4)
	Never married	154 (11.7)
	Separated	23 (1.8)
Education level	Elementary school	454 (34.6)
	High school	303 (23.1)
	Middle school	240 (18.3)
	No formal education	155 (11.8)
	University/college	138 (10.5)
	Graduate school	23 (1.8)
Graduation status	Graduated	979 (74.6)
	Dropped out	177 (13.5)
	No formal education	155 (11.8)
	Completed coursework/on leave	2 (0.2)
Region	Dong (urban area)	949 (72.3)
	Eup/Myeon (rural area)	364 (27.7)
Housing type	Detached house	634 (48.3)
	Apartment	448 (34.1)
	Multi-family house	123 (9.4)
	Row house	74 (5.6)
	Officetel	16 (1.2)
	House in commercial building	15 (1.1)
	Other	3 (0.2)
Type of health security	Employee dependent	553 (42.1)
	Self-employed insured, householder	386 (29.4)
	Employee insured	187 (14.2)
	Medical aid, householder	161 (12.3)
	Self-employed insured, household member	23 (1.8)
	National merit recipient	2 (0.2)
	Medical aid, household member	1 (0.1)
Employment status	Not economically active	625 (47.6)
	Wage worker	326 (24.8)
	Public/support work program participant	186 (14.2)
	Self-employed	166 (12.6)

	Employer	10 (0.8)
Public pension receipt	No	720 (54.8)
	Yes	593 (45.2)
Disability	No	1,142 (87.0)
	Yes	171 (13.0)
Chronic disease	Yes	1,109 (84.5)
	No	204 (15.5)
Need for care	No	1,211 (92.2)
	Yes	102 (7.8)
Regular exercise	Yes	798 (60.8)
	No	515 (39.2)
Lifetime smoking history	Never smoked	930 (70.8)
	≥5 packs (100 cigarettes) lifetime	381 (29.0)
	<5 packs (100 cigarettes) lifetime	2 (0.2)
Average alcohol consumption per drinking occasion	Does not drink alcohol	710 (54.1)
	1–2 drinks	305 (23.2)
	3–4 drinks	128 (9.7)
	7–9 drinks	76 (5.8)
	5–6 drinks	51 (3.9)
	10 or more drinks	43 (3.3)
Perceived stress	Hardly	424 (32.3)
	A little	627 (47.8)
	Much	240 (18.3)
	Very much	22 (1.7)
Subjective health	Very good	28 (2.1)
	Good	333 (25.4)
	Fair	526 (40.1)
	Poor	388 (29.6)
	Very poor	38 (2.9)
Depressive symptoms	No	1,200 (91.4)
	Yes	113 (8.6)
Anxiety	No	1,230 (93.7)
	Yes	83 (6.3)
Suicidal ideation	No	1,256 (95.7)
	Yes	57 (4.3)
Medical aid benefit receipt	No	1,151 (87.7)
	Yes	162 (12.3)
Perceived medical expense burden	Very burdensome	218 (16.6)

	Somewhat burdensome	387 (29.5)
	Moderate	255 (19.4)
	Slightly burdensome	325 (24.8)
	Not burdensome at all	128 (9.7)
Unmet medical needs	Yes	215 (16.4)
	No	1,086 (82.7)
	No need for medical care	12 (0.9)
Unmet dental care	Yes	186 (14.2)
	No	963 (73.3)
	No need for dental care	164 (12.5)
Usual source of care	Yes	996 (75.9)
	No	317 (24.1)
Private health insurance enrollment	Yes	766 (58.3)
	No	541 (41.2)
	Do not know	6 (0.5)

Note. Values are presented as n (%), and all percentages are unweighted. Subjective health is presented using the original survey coding; the reverse-coded subjective health variable was used for model development. Lifetime smoking history was coded according to the KHPS S1 item (<5 packs [100 cigarettes] lifetime, ≥5 packs lifetime, or never smoked).

Supplementary Table S3. Full SHAP ranking of all candidate predictors in the final XGBoost model

Rank	Predictor	Mean absolute SHAP
**1**	Subjective health	0.025178108
**2**	Age	0.015973063
**3**	Walking time (min/day)	0.010587899
**4**	Need for care	0.009951191
**5**	Monthly household income	0.008487012
**6**	Unmet medical needs	0.006007791
**7**	Total annual out-of-pocket expenditure	0.004581106
**8**	Disability	0.004477177
**9**	Anxiety	0.004137004
**10**	Regular exercise	0.003335211
**11**	Employment status	0.003018842
**12**	Chronic disease	0.003017926
**13**	Lifetime smoking history	0.002212195
**14**	Perceived medical expense burden	0.00201685
**15**	Unmet dental care	0.001893124
**16**	Annual inpatient out-of-pocket expenditure	0.001025069
**17**	Annual prescription out-of-pocket expenditure	0.001018805
**18**	Sex	0.000955925
**19**	Annual outpatient out-of-pocket expenditure	0.000817625
**20**	Graduation status	0.000803138
**21**	Housing type	0.000758065
**22**	Education level	0.000652413
**23**	Suicidal ideation	0.000647786
**24**	Usual source of care	0.000609403
**25**	Annual emergency out-of-pocket expenditure	0.000577785
**26**	Depressive symptoms	0.000443174
**27**	Marital status	0.000312547
**28**	Type of health security	0.000246476
**29**	Average alcohol consumption per drinking occasion	0.000142038
**30**	Medical aid benefit receipt	0.000129959
**31**	Perceived stress	0.000115572
**32**	Region	0.000073200
**33**	Public pension receipt	0
**34**	Number of individual Private health insurance enrollment policies	0
**35**	Number of household Private health insurance enrollment policies	0
**36**	Private health insurance enrollment	0

Supplementary Table S4. Sensitivity analysis for structural-zero treatment of cost variables

Analysis	MAE	RMSE	R^2	$\lvert error \rvert \leq 0.05$	$\lvert error \rvert \leq 0.10$
Primary analysis: structural-zero coding for cost variables	0.070	0.096	0.373	0.456	0.764
Sensitivity analysis: median imputation for cost variables	0.070	0.096	0.377	0.452	0.768

Note. The sensitivity analysis used median imputation for cost-variable missing values instead of structural-zero coding. Predictive performance was nearly identical across the two approaches.

Supplementary Table S5. Age-subgroup sensitivity analysis of final model performance

Age subgroup	N_test	Mean age, years	Mean EQ-5D	MAE (95% CI)	RMSE (95% CI)	R^2 (95% CI)	Proportion with |error| ≤ 0.05 (95% CI)	Proportion with |error| ≤ 0.10 (95% CI)
40–64 years	53	53.23 ± 7.44	0.945 ± 0.087	0.038 (0.030–0.048)	0.050 (0.039–0.060)	0.665 (0.430–0.767)	0.755 (0.642–0.868)	0.925 (0.849–0.981)
≥65 years	210	77.13 ± 6.89	0.873 ± 0.125	0.078 (0.070–0.088)	0.104 (0.090–0.120)	0.296 (0.191–0.396)	0.381 (0.314–0.448)	0.724 (0.667–0.781)

Note. The age-subgroup analysis was conducted as a sensitivity analysis using the held-out test set. The final XGBoost model trained on the full training set was applied separately to participants aged 40–64 years and those aged ≥65 years in the test set. Confidence intervals were estimated using 1,000 bootstrap resamples within each subgroup. The younger subgroup had a smaller test-set sample size; therefore, subgroup findings should be interpreted cautiously.

Supplementary Table S6. Top 10 SHAP-ranked predictors by age subgroup

Age subgroup	Rank	Predictor	Mean absolute SHAP	N_test
40–64 years	1	Subjective health	0.022	53
	2	Age	0.0216	
	3	Monthly household income	0.012	
	4	Walking time (min/day)	0.0087	
	5	Need for care	0.0063	
	6	Chronic disease	0.0056	
	7	Unmet medical needs	0.0053	
	8	Anxiety	0.0049	
	9	Total annual out-of-pocket expenditure	0.0045	
	10	Employment status	0.0045	
≥65 years	1	Subjective health	0.026	210
	2	Age	0.0145	
	3	Walking time (min/day)	0.0111	
	4	Need for care	0.0109	
	5	Monthly household income	0.0076	
	6	Unmet medical needs	0.0062	
	7	Total annual out-of-pocket expenditure	0.0046	
	8	Disability	0.0045	
	9	Anxiety	0.0039	
	10	Regular exercise	0.0033	
